# The effect of *Aloe Vera* gel on prevention of pressure ulcers in patients hospitalized in the orthopedic wards: a randomized triple-blind clinical trial

**DOI:** 10.1186/s12906-018-2326-2

**Published:** 2018-09-29

**Authors:** Davood Hekmatpou, Fatemeh Mehrabi, Kobra Rahzani, Atefeh Aminiyan

**Affiliations:** 1 Academic member of Nursing and Midwifery Faculty, Arak University of Medical Sciences, Basij Sq., Payambar-e-Azam Educational Complex, Arak, Iran; 2 Nursing and Midwifery Faculty, Arak University of Medical Sciences, Arak, Iran; 3 Clinical Pharmacology, Academic member of Arak University of Medical Sciences, Arak, Iran

**Keywords:** *Aloe Vera*, Prevention, Pressure ulcer, Orthopedic ward

## Abstract

**Background:**

One of the most common orthopedic problems is the incidence of pressure ulcer followed by immobility. This study aimed to investigate the effect of *Aloe Vera* gel on the prevention of pressure ulcer in patients hospitalized in the orthopedic ward.

**Method:**

This study is a randomized, triple-blind clinical trial which was done on 80 purposefully selected patients in orthopedic ward in Arak town, Iran, 2016. Patients were randomly assigned into two intervention and control groups based on blocking sampling method. In each group the routine daily cares to prevent bed sores were performed by nurses. In the intervention group in addition to routine nursing care to prevent bed sores, twice a day (hours of 9 and 21) pure *Aloe Vera* gel on the areas of hip, sacrum and heel were rubbed, but in the control group placebo (gel of water and starch) were used. Then sacral, hip and heel of both groups on days 3, 7 and 10 for of signs of pressure ulcers was evaluated.

**Results:**

The mean age of patients in the control group was (42.34 ± 12.19) and in the intervention group Was (41.71 ± 11.50) years, respectively. In the intervention group 1 patient afflicted with sore of hip and two people with sacral pressure ulcer. In the control group 3 patients affiliated with sore of hip, 8 people with sacral pressure ulcer, and 1 person had pressure sore of heel. Analysis of the data showed that both groups had statistically significant differences in the incidence of pressure ulcers (*P* = 0.047). This means that *Aloe Vera* gel could prevent the occurrence of pressure ulcers in the intervention group.

**Conclusion:**

Due to the effect of *Aloe Vera* gel to prevent a rise in temperature, non-blanchable redness, swelling and pain of the skin of regions under study in hospitalized patients in the orthopedic ward, applying of it toward the prevention of pressure ulcers in patients at risk of pressure ulcer development is recommended.

**Trial registration:**

This study was registered in Iranian Registry of Clinical. Trials in 07/09/2016 with the IRCT ID: IRCT2016051027825N1.

## Background

A pressure injury is localized damage to the skin and underlying soft tissue usually over a bony prominence or related to a medical or other device. The injury can present as intact skin or an open ulcer and may be painful. The injury occurs as a result of intense and/or prolonged pressure or pressure in combination with shear. The tolerance of soft tissue for pressure and shear may also be affected by microclimate, nutrition, perfusion, co-morbidities and condition of the soft tissue [1]. Pressure ulcers often occur in bone bumps like the sacrum, bumps of ASIS, Heels, trochanter, occipital and shoulder area; it is rarely reported in the nose, ears and lips [1]. Pressure ulcer affects more than 3.1 million adults worldwide annually [2]. The prevalence of pressure ulcers in hospitals in Spain is reported 3.8%, France 9.8%, Germany 2.10%, Portugal 5.12%, Jordan 12%, Ireland 5.18%, Belgium 1.21%, Denmark 22.7%, Sudan, 23%, China 58.1%, Netherlands 3.4%, Austria 0.5%, Switzerland 2.1%, Iran 5% in the public sector and 1.10–21% in the intensive care units [3–6]. It has also a high mortality rate [4], the Emergency Care Research Center of England reported the risk of death in patients with pressure sores 2–6 times more than the other patients [7]. According to statistics in 2012, about 6.1 million patients each year suffer from pressure ulcers in intensive care units, which cost 2.17 billion dollars in America [8] and the annual cost of treatment of pressure ulcers in the UK National Health Service is between 4.1 and 1.2 billion pounds [9]. In addition to incurring high financial expenses for health care institutions, pressure ulcers result in consequences such as loss of credibility of the institution and wasting the valuable time of personnel, and increase the density of nursing staff to 50% [10, 11]. Also this complication increases hospital stay for patients from 4 to 31 days more, it is associated with pain, infection, and complications such as stress, delayed wound healing and impaired mental image [9, 12]. The impact of pressure ulcers on quality of life is significant, considering their influence on physical, psychological, emotional, spiritual, social and financial dimension of life [13].

Most patients admitted to the orthopedic ward are at risk of developing pressure ulcers [14]. Several factors including age, physical limitations, diseases such as diabetes and heart problems, having orthopedic problems, using sedatives, steroids, analgesic and anesthetic, malnutrition, incontinence of urine and feces, the applying change position, having a company like a family member, the use of protective equipment, Fencing of the bed, Wavy Mats (Medical mattress), having traction, the type of it and the use of mobility aids orthopedic sector are involved in the development of pressure ulcers [15]. The principle of care focuses on prevention and is a priority in nursing care [16–18]. Prevention measures of pressure ulcer in accordance with the European Pressure Ulcer Advisory Panel (EPUAP) and National Pressure Ulcer Advisory Panel (NPUAP) 2014 include: Pressure ulcer risk assessment, assessment of skin and skin care, proper nutrition, repositioning, the use of support surface to lower the pressure and patient education on prevention and applying lotions [13, 19–21]. Despite the abundant information on prevention of PUs, it also continues to remain a significant problem in hospital and community. Many researchers have emphasized the importance of reducing the incidence of PUs [9]. Many researchers have emphasized the importance of reducing the incidence of pressure ulcers and have used various methods for prevention and treatment [22]. Also several studies in the field of prevention of pressure ulcers, such as: investigating the effect of olive oil [7], coconut oil [23], the use of sheepskin [24, 25], and hydro-colloid coating [26] had been done.

*Aloe Vera* is a medicinal plant from 1500 years BC in many countries, including Greece, China, Mexico, which had been traditionally used for centuries as a local medicine for various diseases and skin lesions [27]. *Aloe Vera* is an herbaceous and perennial plant with long thick fleshy leaves that belongs to the Liliaceae family and is similar to cactus in appearance [28]. So far, 75 known compounds are found in *Aloe Vera* which contains 20 minerals, 20 amino acids, vitamins and water [29–33]. Among them copper, iron, calcium, zinc, manganese, sodium, potassium, salicylic acid, vitamins A, B, C, E, B12 and folic acid can be pointed out [31]. In vitro studies and in studies that have been conducted on living organisms it has been shown that *Aloe Vera* inhibits thromboxane (an inhibitor of wound healing), helps healing and reduces inflammation [27, 30]. Magnesium lactate in *Aloe Vera* gel prevents the reaction of histamine, which causes itching and irritation to skin [31, 32]. It also enhances the immune system activity and synthesis of the cytokine. By inhibition of IL-6 and IL-8 *Aloe Vera* reduces the adhesion of leukocytes, increases the levels of IL-10 and decreases the levels of TNF alpha, so it is effective in inhibiting inflammatory reactions [33]. Its regenerative property is related to a compound called Glucomannan which is rich in polysaccharides such as mannose that effects on receptors of the fibroblast growth factor and stimulates its activity and proliferation and increases collagen production. *Aloe Vera* gel not only increases the amount of collagen in wounds, but also changes the composition of collagen by increasing the collagen crosslinking and thereby accelerates healing of the wound [34]. Studies show that since 99% of *Aloe Vera* gel is water, it can increase the flexibility of skin and reduce its fragility [28]. Also, the muco-polysaccharides along with amino acids and zinc in *Aloe Vera* help skin integrity, retain its moisture, reduce erythema and help prevent skin ulcers [35]. Many studies have shown that using *Aloe Vera* to treat various wounds such as psoriasis, mouth ulcers, diabetic ulcers herpes and bed sores has had positive effects [28, 29, 36–39].

Given that the care of pressure ulcers is one of the important and challenging issues in medicine and nursing and prevention of ulcers is one of the main tasks of nurses which is also cost-effective, on the other hand, the use of traditional medicine and herbal plants is one of the ways to prevent PUs [40] and *Aloe Vera* has positive effects and benefits on the skin, this study intends to investigate the effect of *Aloe Vera* gel in the prevention of pressure ulcers in patients hospitalized in orthopedic sector.

## Methods

### The study

This is a triple-blind randomized two-group clinical trial to evaluate the effect of *Aloe Vera* gel on the prevention of pressure ulcers in patients hospitalized in the orthopedic ward in 2016. This study was registered in Iranian Registry of Clinical Trials in 07/09/2016 with the IRCT ID: 2016051027825N1. The sample size was determined according to a similar study, including 80 patients assigned to two groups of control and intervention with 40 participants in each (α =0.05, β =0.2, p1 = 0.028, p2 = 0.257)

$$ n=\frac{{\left({Z}_{1-\raisebox{1ex}{$\alpha $}\!\left/ \!\raisebox{-1ex}{$2$}\right.}+{Z}_{1-\beta}\right)}^2\left[{P}_1\left(1-{P}_1\right)+{P}_2\left(1-{P}_2\right)\right]}{{\left({P}_1-{P}_2\right)}^2} $$

In this study the patients, the trained nurse and the statistician did not know anything about the *Aloe-Vera* gel and placebo containers in two intervention and control groups. On the other hand, the data were collected based on the number of each patient to ensure the outcome of blindness assessment.

Inclusion criteria for the study included: willingness to participate in research, lack of skin diseases (such as psoriasis, fungal illness, freckles), age over 18 and under 65 years [8]; patients that are at risk of moderate to severe bedsores according to nursing diagnosis and Braden scoring tool and scored less than 13–14; lack of pressure ulcers on admission [8, 41]; the probability of length of stay should be above 10 days [42, 43]; their admission had been in less than 24 h and had not already been hospitalized in another part [44], lack of systemic diseases such as diabetes, bleeding from trauma, heart failure, kidney failure and cancer advanced phase [9], having a systolic blood pressure of 10 mmHg or higher, not using vasoactive drugs, no drug addiction, no fever (body temperature higher than 38.8), hemoglobin level higher than 12. The Exclusion criteria were: Not wanting to continue to participate in the study, patient death, a decrease in hemoglobin levels during the study to lower than 12 in men and less than 10 mg/dl in women, receiving Vasoactive medications, anemia, reduced pressure, and hyperthermia during the study.

### The study tools

In this study, to collect data a demographic questionnaire was used, as well as Braden pressure ulcer risk assessment scale, and the checklist of daily redness, skin heat, and edema and pain areas.

There are Braden, Waterlow, Norton, and Clinical Judgment tools for assessment of risk of pressure ulcer (Table 1).

**Table 1:** Comparison of Braden, Waterlow, Norton, and Clinical Judgment tools

Assessment tool	Sensitivity (+ True)	Specificity (− True)	Odds Ratio risk prediction	95% Confidence Intervals
Braden Scale	57.1%	67.5%	4.08	2.56–6.48
Waterlow scale	82.0%	27.4%	2.05	1.11–3.76
Norton Scale	46.8%	61.8%	2.16	1.03–4.54
Clinical Judgement scale	50.6%	60.1%	1.69	0.76–3.75

Braden criterion in the clinical judgment of nurses and in the diagnosis of pressure ulcers in comparison with the Waterlow criterion has a higher specificity. On the other hand, Braden criteria can be used in different patients (including patients with acute and chronic ulcers and in different care centers). It requires less time and includes risk factors that are completely objective and easy to use. Sensitivity of Waterlow criterion is high, but its specificity is very low [22, 45]. Based on the Guideline of Wound and Ostomy of Nursing Society (2010), Braden score is a valid tool to assess the risk of pressure ulcer and as a standard questionnaire is widely used in different studies around the world [46].

In Iran to assess the risk of pressure ulcers it is translated and with confirmation its face validity and reliability equal to *r* = 84.1% it has been used [47]. Hence, it is the most common tool used to assess the risk of pressure ulcers. This tool includes six areas of study (sensory perception, moisture, mobility, activity, nutrition and friction force). The scoring is from 6 (highest risk) to 23 (lowest risk) [46]. According to this scale, a score of less than 12 indicates high risk, 13–14 medium risk, 15–16 in patients with 75 years old is low risk and score above 15–18 in patients above 75 years old shows low risk too [11].

The daily record checklist criteria for pressure ulcer include pain, redness, edema, skin temperature of pressure spots in the study. The Checklist has been designed based on the indexes of pressure ulcer grading scale of “National Pressure Ulcer Advisory Panel” [1]. In this study pressure ulcer areas under study (hip, heel, and sacrum) were evaluated and recorded for 10 days.

### Material of the study

Fresh leaves of the *Aloe Vera* littoralis were collected from Ziarat-Ali area (Altitude of sea level is 500 m), Bandar-Abbas, Hormozgan Province, Iran. Voucher specimens of leaves were identified by a botanist scientist (Dr. Mohammad-Amin Soltani Pour) in Hormozgan Province Agricultural and Natural Resources, Research and Education Center, and deposited with the identification number 2229 at the Herbarium Research Center. The leaves were washed before applying.

Starch gel was produced by this way. 30 g of starch purchased from the market was mixed with 70^cc^ of water. Then it was put on a gentle flame. The solution was gently stirred until turn into starch gel. It was used after cooling.

### Implementation of the study

After approval of the proposal, all the hospitalized patients in the orthopedic ward who had the inclusion criteria (targeted sampling) were examined using Braden scale and 80 people who scored less than 13–14 and were at risk of moderate to severe ulcers entered to control and experiment group using random blocking method [First, 16 blocks (*n* = 5) were selected. (Intervention Group = A, Control Group = B) These blocks were sorted by table of random numbers and then allocated into two groups.]. Then PUs prevention methods (e.g. daily evaluation of patient’s skin (every day at 9 am), especially examination of pressure areas such as the sacrum, trochanter, heel, occipital area, shoulder, and changing positions at least every 2 hours, using a small pillow between legs or heels to remove and reduce the pressure, urinary and fecal incontinence control, and using absorbent pads and cleaning the skin in case of contamination) was done on a daily basis by nurses in the form of usual nursing care for patients in both groups. In the experimental group, researchers first tested a part of pure *Aloe Vera* gel on the inner region of forearm and if it does not cause an allergic reaction, it was rubbed (The practice was in this way that the researcher by applying an applicator of *Aloe-Vera* from a dark glass container and rubbing it on the skin of the patient) twice daily (at 9 and 21 o’clock) on pressure points (hip, sacrum, heels) and were given 2–3 min to absorb. The gel was applied for a10 day duration [29]. In the control group the placebo (water and starch gel that was quite similar to *Aloe Vera* gel and was in a glass container which was the same in both groups) were used. Where the researcher treated the same areas (as in the intervention group) in the control group with placebo and then dried the skin with paper towels from the placebo. For drying, a tissue was put and removed and pulling the skin was avoided.

As a matter of fact, both gels rubbed on the patients’ skins (in intervention and control groups) without any forces pressure or massage on the skin. Of course, in the control group, starch gel was removed immediately to prevent starch absorbing or its cooling effect on the skin.

**Fig. 1:**
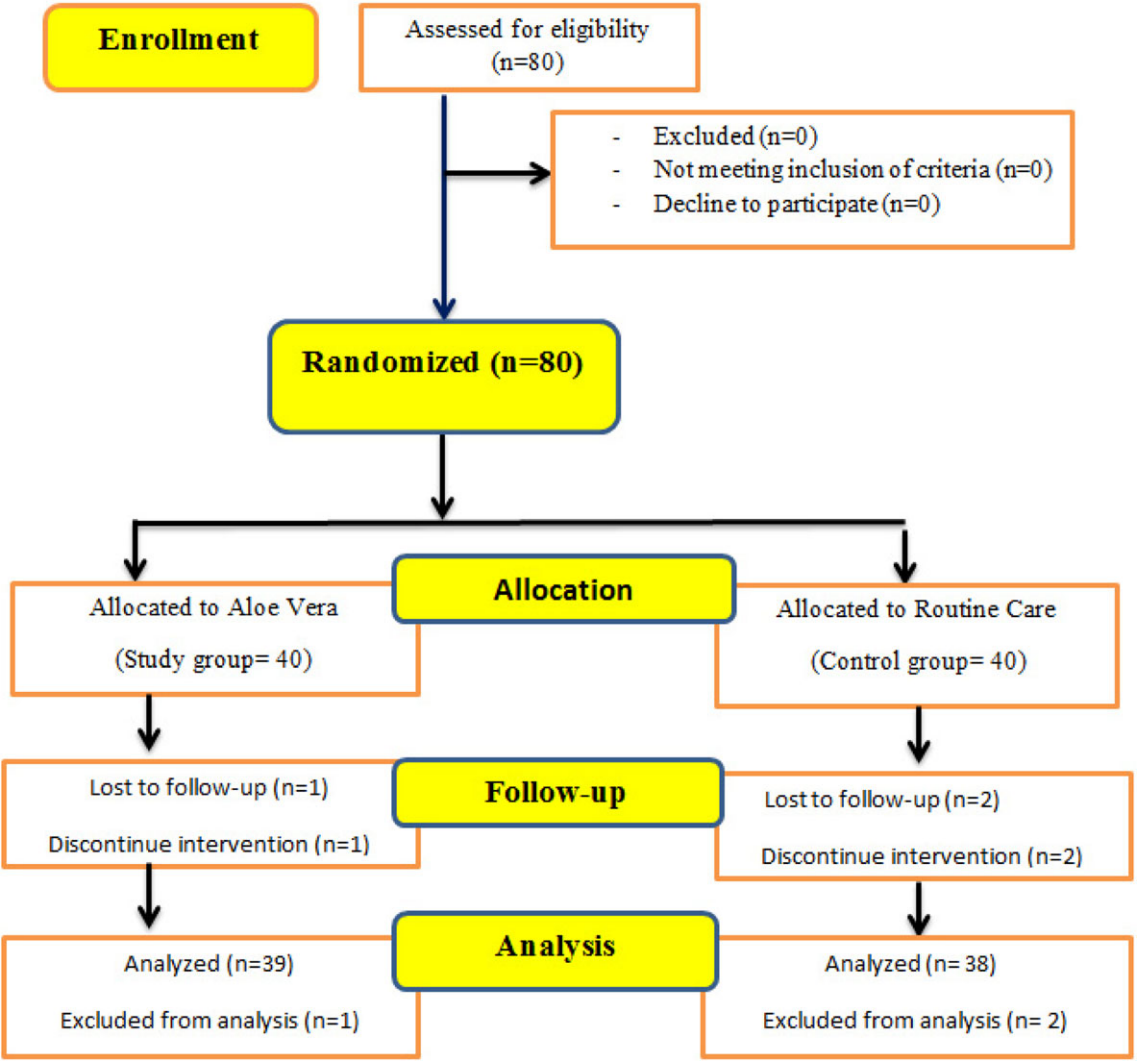
Consort flow diagram shows who the patients enrolled in the study

Then, the sacrum, hip (trochanter), and heel of both groups of patients were examined on days 3, 7 and 10 in terms of presence or absence of signs of pressure ulcers based on the indexes (Fig. 1).

The pressure ulcer index on areas studied was completed every day before and after 9 am by a trained nurse using Microlife infrared thermometer made in Switzerland to measure the skin temperature and observation of other indexes. Any increase in temperature and persistent redness which was indelible by finger, as well as localized swelling, edema and pain in the sacrum, hip, and heel in both groups was considered as local inflammation and pressure ulcers stage 1 [1].

To obtain pure *Aloe Vera* gel, after washing and drying the leaves, the middle mucilage was separated such as fish fillets and was used. The obtained mucilage was transparent non-sticky and odorless, and had a high absorption [26, 40].

### Statistical methods

In this research, SPSS 21 software was used to analyze the data. Descriptive statistics were used to describe the data and to extract the tables and charts. Chi-square tests, Fisher’s exact test, independent t-test, ANOVA with repeated measures, and Friedman were used for interpreting the data in inferential statistics.

### Ethical considerations

This study was planned in Medical Ethics committee of Arak University of Medical Sciences in 19 June 2016. After confirmation, the ethical code of IR.ARAKMU.REC.1395.40 was given to it. This study was done based on all instructions of ethical codes of Tehran declaration of Ethics in Medical Research.

## Results

Of 80 patients, 38 patients in control group and 39 in intervention group were remained. Three of them (2 patients from control group and 1 patient from intervention group) were excluded. Analysis of the data showed that both intervention and control groups had no statistically significant difference in terms of demographic features and other variables before intervention (*P* > 0.05), which means that the two groups were homogenous before the intervention (Tables 2 and 3).

**Table 2:** Frequency of demographic variables of two groups

Variables		Intervention	Control	*P*-value
Age (Mean /SD)		11.50 ± 41.71	12.19 ± 42.34	^a^*t* = − 0.231, df = 75, PV = 0.512
Sex (Male %)		71.8%	73.7%	PV = 0.169^b^
Braden score (Mean)		1.50 ± 11.79	1.27 ± 11.81	^a^*t* = − 0.066, df = 75, PV = 0.150^a^
Type of orthopedic injury	Hip fractures	10 (25.6%)	11 (28.9%)	^b^PV = 0.932
	Femoral head fracture	12 (30.8%)	13 (34.2%)	
	Pelvic fracture	3 (7.7%)	2 (5.3%)	
	Vertebral fractures	2 (5.1%)	3 (7.9%)	
	Multiple trauma(Fractures in several areas)	12 (30.8%)	9 (23.7%)	

^a^Independent t-tests

^b^Fisher’s exact test

**Table 3:** Frequency of Pressure Ulcers among two groups

variables		Intervention	Control	*P*-value
Number of PU per patient		2 (5.1%)	8 (21.1%)	*t* = 4.815, df = 1, PV = 0.047
Anatomical site of PU	Hip(trochanter)	1 (1.3%)	3 (3.9%)	
	Sacrum	2 (2.6%)	8 (10.4%)	
	Heels	0 (0)	1 (1.3%)	

### Hip (trochanter) temperature

**Fig. 2:**
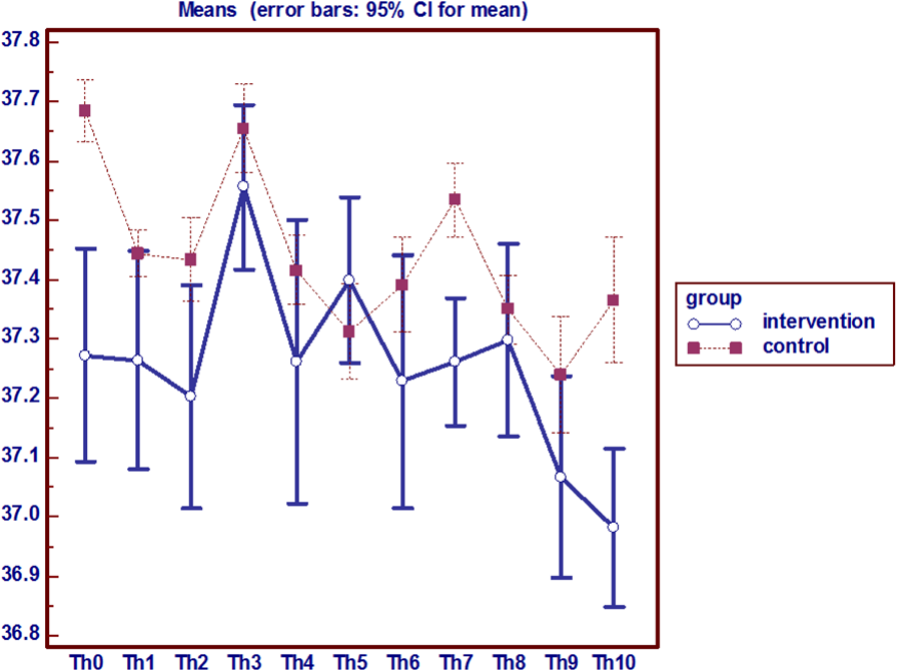
Analysis of variance with repeated measures on the hip (trochanter) temperature

Analysis of variance with repeated measures on the hip temperature indicator in two groups shows that from the baseline (first day) to the end of the study (day 10) the average hip local temperature in the intervention group was lower than the control group; and a significant difference was observed between the two groups in the seventh and tenth day (*P* = 0.0001) (Fig. 2).

### Hip (trochanter) redness, edema and pain

Frequency of non-blanchable redness in the hip was 1 in the intervention group and 3 people in the control group. Also, hip edema has also started on the eighth day, in 1 person in the intervention and 2 in the control group. The study results showed that the local hip pain had not existed till the seventh day in the intervention group and is lower than the control group. On the other hand, the process of pain in the control group had been increasing opposed to the intervention group (*P* = 0.003).

**Fig. 3:**
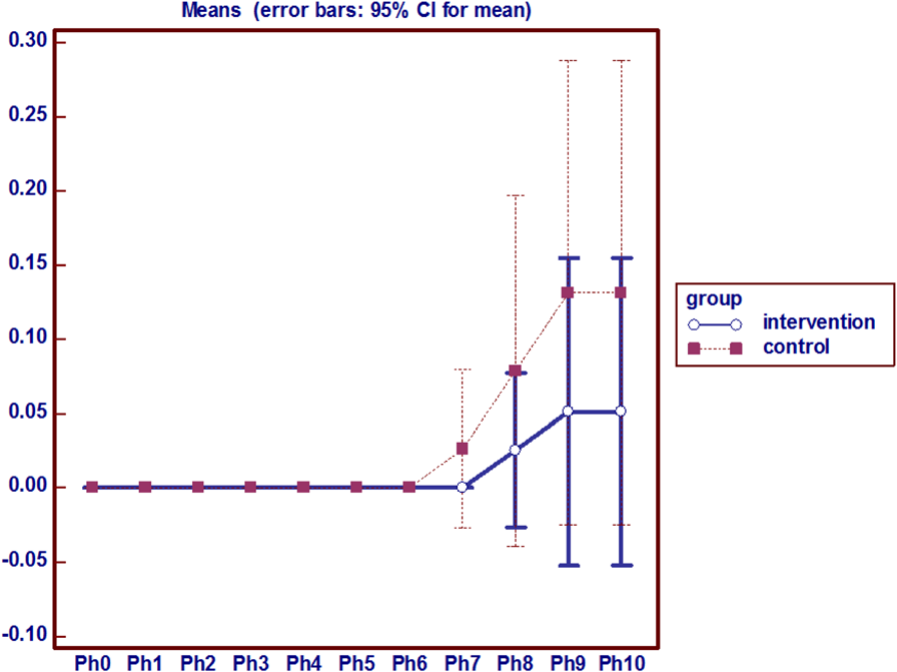
Analysis of variance with repeated measures on the hip (trochanter) Pain

In general, according to the temperatures, persistent and indelible redness, pain and edema, it can be said that on the eighth day of the study 1 subject in the intervention group and 3 patients in the control group get involved with pressure ulcers on the hip (Fig. 3).

### Sacrum temperature

**Fig. 4:**
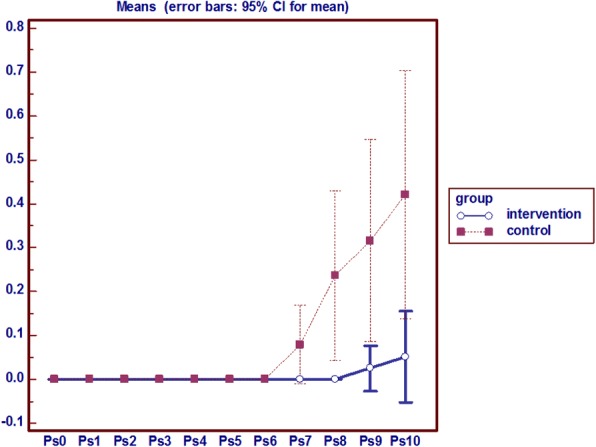
analysis of variance with repeated measures on the temperature of Sacrum

Analysis of variance with repeated measures on the temperature indicator shows sacrum average temperature after the intervention at different times had a statistically significant difference in the two groups (*p* = 0.0001) and sacrum average temperature was lower in the intervention group than the control group (Fig. 4).

### Sacrum redness, edema and local pain

**Fig. 5:**
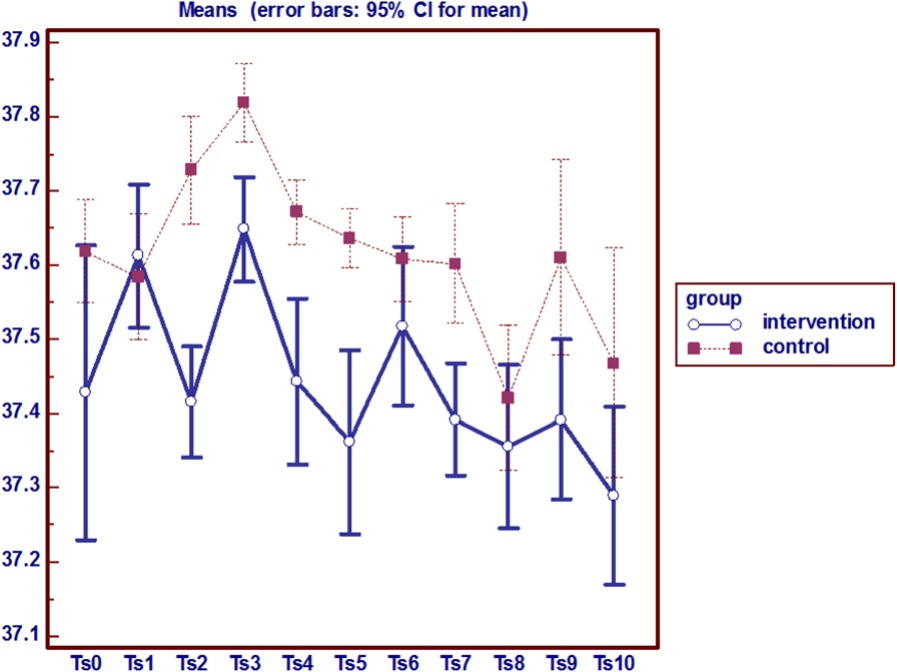
Analysis of variance with repeated measures on the Pain of Sacrum

In the intervention group on the eighth day onward, sacrum redness was seen in 2 patients, which had a constant process, but in the control group the sign emerged from the sixth day onwards among 8 people incrementally. In the experimental group there was no edema in the sacral region during the intervention, but in the control group sacral edema were observed in 5 subjects from the seventh day onwards. Pain in the control group started from the seventh day and from the ninth day in the study group. Mann-Whitney test results show that the average sacral pain in the intervention group had a statistically significant decrease during the eighth, ninth and tenth day compared to the control group. Friedman test results also showed that the average sacral pain in the intervention group had a roughly constant process but at the control group increased from the seventh day onwards (*p* = 0.001) (Fig. 5).

In general, according to the observed temperature, persistent redness, pain and edema, it can be said that 2 patients in the intervention group since the eighth day and 2 patients in the control group since the sixth day were affected with pressure ulcers in the sacrum area.

### Heel temperature

**Fig. 6:**
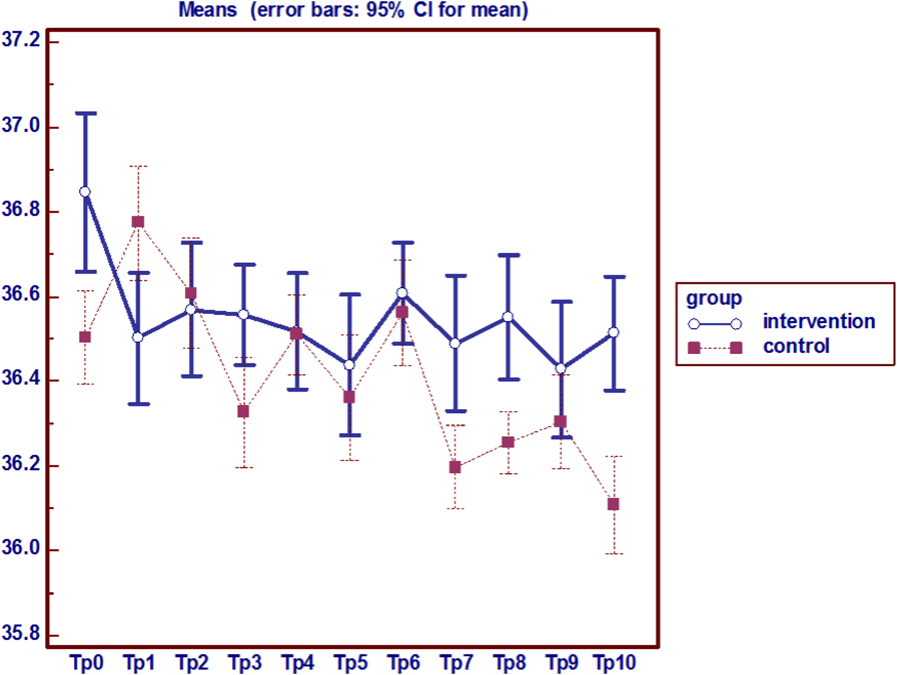
Analysis of variance with repeated measures on the heel temperature

Analysis of variance with repeated measures on the heel temperature index in two groups showed that the average temperature in the heel after the intervention at different times had a statistically significant difference in the two groups (*p* = 0.0001). The average temperature in the heel was higher in the intervention group than the control group and the difference was statistically significant after the intervention at different times in the two groups (*p* = 0.0001) (Fig. 6).

### Heel redness, edema and local pain

**Fig. 7:**
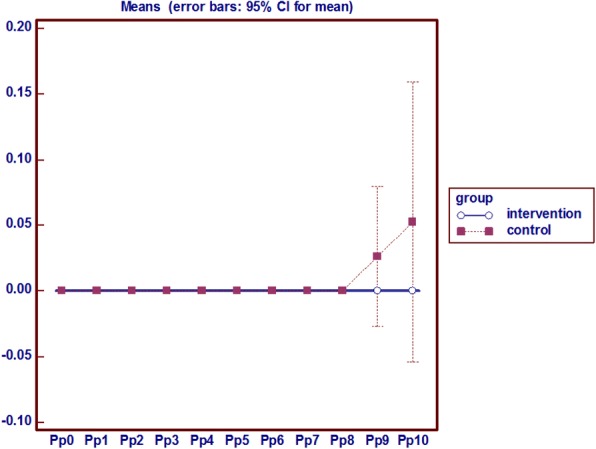
Analysis of variance with repeated measures on the heel pain

The persistent non-blanchable redness of heel was observed only in 1 patient in the control group since the ninth day, however no indelible redness and pain was observed the intervention group. Also, no edema was observed in this area in both groups (Fig. 7).

The results of temperature changes, persistent non-blanchable redness, edema and local pain indicates that no subject in the intervention group and 1 subject in the control group were affected by pressure ulcer stage 1 in the heel area.

During the intervention it was observed that on the basis of pressure ulcer indexes of EPUAP/NAPUAP [3] 1 patient in the intervention group get involved with hip pressure ulcer and two people with sacrum pressure ulcers, whereas in the control group 3 patients were affected with hip ulcer, 8 patients with sacrum pressure ulcer and one with heel ulcer (Table 4).

**Table 4:** Frequency of pressure ulcers in the areas under study

The incidence of pressure ulcers	pressure ulcers of hip (trochanter)	pressure ulcers of sacrum	pressure ulcers of heel	Total
frequency^a^	Number/ Percent	Number/Percent	Number/Percent	
intervention	1 (2.5)	2 (5.1)	0 (0)	3
control	3 (7.8)	8 (21)	1 (2.6)	12
total	4 (10.3)	10 (26.5)	1 (2.6)	15

^a^(In some cases, there was pressure ulcers occurrence in more than one area)

Analysis of the data showed that the intervention and control groups had statistically significant differences in terms of the incidence of pressure ulcers by intervention (*p* = 0.047). This means that *Aloe Vera* gel could prevent the occurrence of pressure ulcers grade 1 in the intervention group (Table 5).

**Table 5:** Frequency of pressure ulcers in two groups

Groups	Intervention	Control	t-test/ *P*- value
Variable	Number/ Percent	Number/ Percent	
Pressure ulcers	2 (5.1)	8 (21.1)	*t* = 4.319, df = 1, PV = 0.047

## Discussion

The European Pressure Ulcer Advisory Panel (EPUAP) and National Pressure Ulcer Advisory Panel (NPUAP) states that to detect pressure ulcers, skin observation should include an assessment of temperature changes, the incidence of persistent indelible redness, swelling or stiffness and pain and tenderness in the region [1]. According to this point, we can say a temperature rise at the hip and sacrum in the control group and decrease in the temperature in the heel in the control group demonstrated the incidence of pressure ulcers. In other words, the intervention of using *Aloe Vera* gel on the hip, sacrum and heel of patients had been effective and prevented temperature changes that reflect the occurrence of pressure ulcer. In various studies, the localized temperature changes (both increase and decrease) are introduced as a predictor of pressure ulcer symptoms, especially the grade one ulcer [48–53]. Sae-Sia et al. (2007) state that body temperature is one of the important signs for the prevention and identification of pressure ulcers. In this study, patients who have had orthopedic trauma had an increase of temperature due to the systemic inflammation and the local inflammation of the skin under pressure [53]. Kottner et al. (2015) consider local skin temperature change (especially the temperature rise) and discoloration of the skin in areas under pressure as the most important signs of pressure ulcers [54].

Another sign of pressure ulcer is pain, which was more in the examined areas in the control group than in the intervention group, which is consistent with the other studies [54–58]. Pain in pressure ulcers is caused by damage to the nerves and local inflammation [57]. Briggs et al. (2013) reported the prevalence of pressure ulcer pain 3.16% [57]. While Gorecki et al. (2011) report the prevalence of pressure ulcer pain in the 5.70% and in subjects who are at risk of ulceration 6.12% [55, 57, 59]. Girouard et al. (2008) believe that pain is associated with an increased degree of pressure ulcers. However, factors such as age, underlying diseases, scars and pressure affect the intensity of pain [60]. But Mac Genesis (2014) and Briggs (2013) consider pain intensity independent from the degree and intensity of pressure ulcers [56, 58]. In general, mild pain appears in the first and second stage ulcer and severe pain in the ulcer grade four onwards [61]. In this study, a mild pain emerged in pressure ulcer degree 1 and is more in the control group than in the intervention group. Pain reduction in the intervention group could be due to the effects of the use of *Aloe Vera* in the areas under study, previous studies have also confirmed the analgesic and anti-inflammatory properties of *Aloe Vera* and is able to reduce pain in the areas under study [29, 62].

Permanent and indelible redness which doesn’t turn white with finger pressure is the most important symptom of pressure ulcer that has long been used. In the past, by the study of redness, pressure ulcer degree one was diagnosed. But now, attention to both visible symptoms (swelling and redness) and non-visible ones (local temperature, pain, tissue integration) is necessary [1, 48, 49]. If the skin discoloration is associated with other non-visible signs, it confirms the pressure ulcer [54, 63]. In this study redness in the control group was more than in the intervention group, which represents the effect of *Aloe Vera* gel in the prevention of redness and pressure ulcers in the intervention group. The results are consistent with the study by Sprigle [49].

In present study, the frequency of hip and sacrum local edema emerged less and later in the intervention group compared to the control group and edema of the heel had not emerged completely. Regardless of the degree of edema, it is the sign of pressure ulcer grade one. Pressure on the area, causes inflammation and changes in vascular permeability and face edema [61].

According to the assessment of temperature, edema, non-blanchable redness and pain in areas under study, results indicate that in the tenth day pressure ulcer appeared in the hip (1 person in the intervention group and 3 patients in the control group), in the sacrum region (2 patients in the intervention group and eight patients in the control group), and in the heel area (no one in the intervention group and 1 in the control group).

According to the EPUAP/NPUAP(2014), definition of pressure ulcer grade one include: healthy skin with a pale red area or a different color that is usually on the bone protruding; that may be painful,, smooth, soft, warmer than adjacent tissue. In this type of wound, persistent indelible redness with increased edema (regardless of grade, width and depth) and temperature and pain and tenderness are considered the symptoms of pressure ulcer [61, 64, 65]. Based on the mentioned points, we can say that eight patients in the control group and only two patients in the intervention group had redness, temperature changes, edema and pain in areas under study or in other words, were affected by ulcer grade one. At the end of the study, the two groups had statistically significant differences in the incidence of pressure ulcers and the hypothesis of the study maintaining that the incidence of pressure ulcers is not different in the intervention and control groups after the intervention is rejected. Thus, the intervention was effective in this area, and could prevent ulcer pressure especially grade one ulcer. As mentioned earlier, *Aloe Vera* is anti-inflammatory, antibacterial, antiviral, and antiseptic, protects the skin, heals and prevent wounds that has been mentioned in several studies [29]. The results of the present study are consistent with results of other studies [66–70]. In this study, the use of *Aloe Vera* could help prevent the occurrence of pressure ulcers. Lopianz Perez et al. (2013) and Behnam Moghaddam et al. (2017) found the use of olive oil and Dhikhil et al. (2013) found the use of coconut oil effective in this regard [5, 23, 70]. Prophylactic effect of *Aloe Vera* in healing is because of mucopolysaccharides with amino acids and zinc found in *Aloe Vera* that retains the integrity of the skin, its moisture and reduce erythema and helps to prevent skin ulcers [35]. West et al. (2003) study confirms the results of present study [35]. *Aloe Vera* has been effective in chronic wounds such as pressure ulcers, diabetic ulcers, chronic anal fissure wounds, chronic wounds caused by accidents, psoriasis, genital herpes and acute wounds including burn wounds and surgery wounds such as episiotomy and cesarean, skin biopsy, Hemorrhoidectomy, gynecologic surgery laparotomy and the graft. In this regard, several studies were reviewed and it was observed that the effect of *Aloe Vera* had been higher compared with the current treatments [30, 36, 68, 71, 72] and only one study has suggested that the difference between the two groups was not significant that is due to the low number the samples compared with other studies [68]. In these studies, *Aloe Vera* reduced pain, bleeding and recovery time; there was no infection in the wound, no redness and itching.

Based on our findings sacrum and heel showed the most and least pressure ulcer occurrence, respectively; in other studies the most common spots involved are reported first the sacrum, then the heel and the hip [2, 5, 42, 44, 73–75].

For ulcer prevention and wound healing, *Aloe Vera* is much more effective and less costly compared to other current treatments and since the revival of traditional medicine is important and the side effects of this drug has been proven to be trivial over the years, it seems *Aloe Vera* is a good substitute to replace the current methods or to be used as a complementary method for prevention of pressure ulcers. In general, it could improve the prevention of pressure ulcers and improve community health. Since the numbers of samples were limited, doing similar studies on more samples is recommended.

## Conclusion

At the end, the two groups had statistically significant differences in terms of pressure ulcer occurrence. So it can be said that the intervention was effective in this area, and could prevent the occurrence of pressure ulcer grade one. *Aloe Vera* is much more effective and less costly in the prevention and healing the ulcers compared to current treatments. Also, since the revival of traditional medicine is important and the side effects of this drug has been proven to be trivial over the years, it seems *Aloe Vera* is a good substitute to replace the current methods or to be used as a complementary method for prevention of pressure ulcers and improving community health.
